# Heart rate variability and plasma nephrines in the evaluation of heat acclimatisation status

**DOI:** 10.1007/s00421-017-3758-y

**Published:** 2017-11-10

**Authors:** Major Michael John Stacey, S. K. Delves, D. R. Woods, S. E. Britland, L. Macconnachie, A. J. Allsopp, S. J. Brett, J. L. Fallowfield, C. J. Boos

**Affiliations:** 1 Department of Surgery and Cancer, Imperial College London, Care of General Intensive Care Unit, Hammersmith Hospital, Du Cane Road, London, W12 0HS UK; 2 Department of Military Medicine, Royal Centre for Defence Medicine, ICT Building, Birmingham Research Park, Vincent Drive, Edgbaston, Birmingham, B15 2SQ UK; 3 Institute of Naval Medicine, Alverstoke, Hampshire, PO12 2DL UK; 4 Carnegie Research Institute, Leeds Beckett University, Leeds, LS6 3QS UK; 5 Cardiology Department, Poole Hospital NHS Foundation Trust, Poole, BH15 2JB UK

**Keywords:** Autonomic nervous system, Catecholamines, Normetanephrine, Metanephrine, Heat adaptation, Heat illness, Heat stroke

## Abstract

**Purpose:**

Heat adaptation (HA) is critical to performance and health in a hot environment. Transition from short-term heat acclimatisation (STHA) to long-term heat acclimatisation (LTHA) is characterised by decreased autonomic disturbance and increased protection from thermal injury. A standard heat tolerance test (HTT) is recommended for validating exercise performance status, but any role in distinguishing STHA from LTHA is unreported. The aims of this study were to (1) define performance status by serial HTT during structured natural HA, (2) evaluate surrogate markers of autonomic activation, including heart rate variability (HRV), in relation to HA status.

**Methods:**

Participants (*n* = 13) were assessed by HTT (60-min block-stepping, 50% *V*O_2_peak) during STHA (Day 2, 6 and 9) and LTHA (Day 23). Core temperature (Tc) and heart rate (HR) were measured every 5 min. Sampling for HRV indices (RMSSD, LF:HF) and sympathoadrenal blood measures (cortisol, nephrines) was undertaken before and after (POST) each HTT.

**Results:**

Significant (*P* < 0.05) interactions existed for Tc, logLF:HF, cortisol and nephrines (two-way ANOVA; HTT by Day). Relative to LTHA, POST results differed significantly for Tc (Day 2, 6 and 9), HR (Day 2), logRMSSD (Day 2 and Day 6), logLF:HF (Day 2 and Day 6), cortisol (Day 2) and nephrines (Day 2 and Day 9). POST differences in HRV (Day 6 vs. 23) were + 9.9% (logRMSSD) and − 18.6% (logLF:HF).

**Conclusions:**

Early reductions in HR and cortisol characterised STHA, whereas LTHA showed diminished excitability by Tc, HRV and nephrine measures. Measurement of HRV may have potential to aid real-time assessment of readiness for activity in the heat.

**Electronic supplementary material:**

The online version of this article (10.1007/s00421-017-3758-y) contains supplementary material, which is available to authorized users.

## Introduction

Heat adaptation (HA) is critical to human health and performance during endurance-related physical activity performed under heat stress. Effective HA can augment exercise capacity ‘into realms within which physiological intolerance and systemic failure might otherwise be observed’ (Taylor 2014), whereas ineffective HA may contribute to significant debility from heat illness, or even death from heat stroke (Bouchama and Knochel 2002).

For athletes training and competing in the heat, HA has been presented as ‘the most important intervention … to reduce physiological strain and optimise performance’ (Racinais et al. 2015). Among individuals exposed to recreational heat stress, such as hikers and mountain-bikers, seasonal variation in HA status may account for higher rates of heat-related illness at the onset of hot summer weather (Noe et al. 2013). The challenge of maintaining performance and protecting health in conditions of higher heat stress is also faced by other groups, such as military personnel, fire fighters, miners, hazardous waste disposal operators and agricultural workers, who may have occupational cause to move rapidly from a less to more severe environment.

HA is promoted by daily exercise bouts in artificial environments (heat acclimation) or hot natural climates (heat acclimatisation). Short-term heat acclimatisation (STHA) occurs during the first days of exercise in the heat, with a shift in homeostatic mechanisms towards promoting heat dissipation (Periard et al. 2015). Traditional investigative approaches show progressive physiological changes when exposures are prolonged beyond 7–14 days (Tyler et al. 2016), resulting in ‘maintenance of lower body temperature, lower pulse rate and readier sweating’ (Adolph 1947). Exposures lasting ≥ 3 weeks develop cytoprotective adaptations to thermal stress and result in the phenotype of long-term heat acclimatisation (LTHA). The epigenetic adaptations associated with LTHA—but not STHA—have been reported to attenuate initial cellular damage from thermal injury and other stressors; accelerate spontaneous recovery from these insults; and potentially enhance endurance performance due to increased end organ efficiency under heat stress (Horowitz 2014, 2016).

The autonomic nervous system (ANS) is central to cardiovascular regulation during and after exercise bouts (Iellamo 2001; Parekh and Lee 2005; Armstrong et al. 2012). In animal models subject to chronic heat stress, ANS excitability measured ex vivo diminishes with the transition from STHA to LTHA (Horowitz and Meiri 1993; Horowitz 2014, 2016). During exercise performed under thermal stress, the ANS buffers increased competition for blood flow between skeletal muscle and the cutaneous circulation (Rowell 1990). Observable measures of ANS disturbance include heart rate variability (HRV) (van Ravenswaaij-Arts et al. 1993)—which describes the fluctuation of instantaneous heart rate over time (Heathers 2014)—and indicators of sympathetic activity such as catecholamines and their nephrine metabolites (Raber et al. 2000; Bracken et al. 2009; Bracken and Brooks 2010).

The initial few minutes of recovery following exercise are a favourable period for assessing autonomic activity, as the level of ANS activation may be characterised using HRV (Michael et al. 2016). In response to repeated submaximal exercise bouts in the heat, norepinephrine levels measured within 60 s of exercise termination have been reported to decline over an 8 day protocol, with the largest reduction occurring over the first 3 days (Hodge et al. 2013). Circulating catecholamines have very short (1–2 min) half-lives, however, such that the potential for measurement to be confounded by preceding changes in posture and the stress of venepuncture (Peaston and Weinkove 2004; Raber et al. 2000) may favour assay of their more stable extraneuronal nephrine metabolites (normetanephrine and metanephrine) in the assessment of sympathoadrenal activation (Peaston and Weinkove 2004; Raber et al. 2000). The potential value of plasma nephrines in discriminating the level of extrinsic stress experienced during standardised exercise bouts has been highlighted, with variation in normetanephrine levels reflecting the nature of environmental challenge faced (Woods et al. 2017).

Consensus guidelines recognise that individual HA responses are highly variable and recommend personalised HA strategies, to increase work capacity and reduce the risk of heat illness (Bergeron et al. 2012; Racinais et al. 2015). Objective ascertainment of HA progress is recommended, by assessment of physiological responses to exercise in the heat (Bergeron et al. 2012). Standardised heat tolerance test (HTT) has been endorsed for validating HA status in athletes (Racinais et al. 2015; Karlsen et al. 2015) and military personnel (Taylor 2014), but relies on assessing conventional physiological responses (i.e. heart rate and core temperature) that may reflect whole-organism strain and resistance to thermal injury incompletely.

Data on how HRV and biochemical surrogates for ANS activity vary with HA are limited, but point to increased parasympathetic activity and reducing sympathetic tone with progressive adaptation (Horowitz and Meiri 1993; Flouris et al. 2014). Concurrent changes in these parameters have not been described in relation to HTT or during HA. Furthermore, any role for these parameters in distinguishing STHA from LTHA is unreported. As it has been argued that the transient condition of STHA drives phenotypic changes towards a more efficient, stable and protective state (LTHA) over time (Horowitz 2014), there may be a potential role for HRV identifying such ANS perturbations as precede the acquisition of advanced heat tolerance (Assayag et al. 2010, 2012).

Thus, the aims of this study were to (1) define physical performance status during HA, using serial HTT and (2) evaluate surrogates of autonomic activation, including HRV and blood nephrine concentrations, as potential markers of HA status.

## Methods

### Volunteers

The study was approved by the United Kingdom (UK) Ministry of Defence Research Ethics Committee and complied with the standards set in the Declaration of Helsinki (Fortaleza; protocol number 531/MoDREC/14). Eighteen male military personnel were recruited to the study in the UK in May 2014. Volunteers were drawn from UK-based military units that had not deployed to a hot climate during the preceding 6 months. Volunteers were required to complete a health history questionnaire and pass a medical assessment with the study Independent Medical Officer. Volunteers had no prior history of heat illness and were not taking vasoactive or psychotropic medication. Volunteers abstained from alcohol for 24 h before all study measures. All volunteers gave written informed consent.

### Study design

The study was completed in two phases (see online Appendix). During Phase I, volunteers attended a UK laboratory (Institute of Naval Medicine, southern England) for medical assessment, detailed baseline measurements and study measures, including familiarisation HTT. They spent the following week taking part in directed military training locally, then deployed en bloc to the Mediterranean island of Cyprus for Phase II of the study. This consisted of structured heat acclimatisation and directed military training, closely matched to that conducted in the UK. Four HTTs were conducted in the same climatic chamber during STHA (Day 2, Day 6, Day 9) and LTHA (Day 23).

### Phase I

Measurements were taken during morning hours. First, medical assessment was conducted, including exclusion of significant dehydration by urinary specific gravity. Next, volunteer height (± 0.01 m), body mass (± 0.001 kg) and body composition (Tanita MC 180MA Segmental Multi Frequency Body Composition Monitor Class III; Tanita UK Ltd., Yiewsley, Middlesex, UK) were recorded. Assessment of peak oxygen uptake (*V*O_2_peak) was then performed in a climatic chamber maintained at WBGT 27.3 ± 0.1 °C. For *V*O_2_peak assessment, volunteers were asked to run on a treadmill following a standardised ramp protocol, starting at 8 km h^−1^, whereupon reaching 13 km h^−1^ the gradient was increased by 2% every minute. Expired gas and gas volume was measured by online metabolic cart (Cosmed, Quark b^2^, Rome, Italy) to determine peak oxygen consumption (peak *V*O_2_) at the point of volitional exhaustion, whereupon the assessment was terminated. Volunteers were rested in a cool environment for ≥ 60 min, before returning to the chamber to undertake HTT.

### HTT protocol

Volunteers entered the climatic chamber on foot immediately upon completion of PRE measures, where they rested in a standard chair for 30 min. They then performed 60 min of stepping exercise, on/off a 0.32 m-high block. In the UK, the relative exercise intensity was adjusted during the first 5 min to 50% *V*O_2_peak. Stable absolute oxygen consumption was confirmed on three successive measurements. Adjusted stepping rate was maintained thereafter and during all subsequent HTTs, with the use of an in-ear metronome (Seiko SQ50, Seiko Instruments, Chiba, Japan) adjusted to the appropriate cadence. Stepping was performed under direct supervision of observers positioned inside the chamber. Immediately after each HTT, excess sweat was towelled from volunteers’ skin. They then returned to the adjacent room for POST measures.

### Phase II

In Cyprus, standard UK Ministry of Defence (MoD) guidance on HA was followed (https://www.gov.uk/government/publications/prevention-of-climatic-injuries-in-the-armed-forces-medical-policy). This consisted of graded exposure to exercise outdoors in the heat, with increasing levels of military dress and load carriage. HTTs were substituted in place of programmed acclimatisation exercise on Day 2, 6, 9 and 23. HTT was conducted in a climatic chamber, during morning hours and at the same time of day for each individual assessed. The environment inside the chamber was maintained at dry bulb 33.6 ± 0.5 °C, wet bulb 23.5 ± 0.3 °C; Globe 33.8 ± 0.4 °C; WBGT index 26.6 ± 0.3 °C. Volunteers were instructed to refrain from all eating and drinking from point of arrival for study measures pre-HTT, until after completion of measurements post-HTT. This meant that HTT was performed without replacement of fluid losses.

Outside of HTT assessments, volunteers lived and worked in natural ambient conditions in and around a military garrison, without access to air conditioning, but with ample access to food, drinking water and beverages that could be taken ad libitum. From Day 10 onwards, volunteers were at liberty to perform physical exercise in addition to programmed activities (i.e. outside of working hours and on allocated rest days). Environmental conditions were recorded by WBGT monitors (Grant, Cambridge, UK) stationed inside the climatic chamber and outside the study facility.

### PRE and POST measures

Volunteers were prepared for HTTs and underwent study measures before (PRE) and afterwards (POST), in a room adjacent to the chamber. Volunteers were rested for 30 min on arrival. They voided and were weighed nude, redressed and commenced PRE sampling. This began by resting in a standard chair for a period of 60 s, after which HRV was recorded continuously for 5 min. Volunteers were instructed to remain still in the chair and refrain from talking throughout. Blood was then venesected from an antecubital fossa vein into a serum separator tube (serum cortisol) and EDTA tube (plasma free metanephrines). POST sampling was performed in the same manner as PRE, with HRV recorded from 3 min and blood sampled from 8 min following HTT. Volunteers were again weighed nude, before being allowed to eat and drink.

### Intra-HTT measures

Volunteer core body temperature (Tc) was monitored during HTT by radiotelemetry pills and paired data loggers (VitalSense, Mini Mitter Company Inc, Oregan, USA), with factory calibration (± 0.01 °C) of individual pills confirmed by water bath the day prior. Volunteers were provided with a pill to swallow 2 h before arrival to the testing facility. Pill-ingestion and adequate temperature logging were confirmed on arrival. Volunteers were then fitted for continuous ambulatory heart rate recording by three lead ECG (Lifecard 12; Spacelabs Healthcare, Hertford, UK), sampling at a rate of 128 Hz. Core body temperature, heart rate and relative perceived exertion (RPE) (Borg 1970) were recorded manually every 5 min during the HTT.

### HRV analysis

Continuous analysis of heart rate was undertaken using a three lead ambulatory ECG. Analysis of HRV was performed by a technician blinded to the study design, using the Pathfinder Ambulatory ECG analysis system described previously (Chow et al. 2014). HRV was obtained over a 5-min segment of successive normal RR (NN) intervals. Time domain analysis was used to assess parasympathetic modulation of the heart, by quantification of the root mean square of successive differences between NN intervals (RMSSD). Spectral analysis was performed on each segment, using the Fourier transformation, to determine the ratio of low-frequency power (LF, range 0.04–0.15 Hz band) to high-frequency power (HF, 0.15–0.4 Hz band).

### Biochemistry

Blood samples were stored in ice and centrifuged within 1 h of collection. Serum and plasma were then frozen to − 20 °C and transported back to the UK in dry ice, where they were frozen to − 80 °C until analysis. Samples were processed by a supra-regional specialist endocrine laboratory (Royal Victoria Infirmary, Newcastle Upon Tyne). Plasma free nephrines (nometanephrine and metanephrine) were measured using an in-house liquid chromatography/tandem mass spectrometry method (co-efficient of variation 4–12%). Serum cortisol was measured by competitive immunoassay (Roche) using fully automated electrochemiluminescence technology (functional sensitivity < 8.5 nmol L^−1^).

### Sample size

A power calculation was not performed prospectively as the sample size was dictated by the availability of equipment and personnel and the capacity of the climatic chamber. It was noted, however, that a sample of 11 responders to 8 weeks of running training showed a significant trend of increasing RMSSD between the start and the end of that programme (Buchheit et al. 2010). Furthermore, a sample of 15 soccer players was adequate to demonstrate a significant increase in a closely related HRV index following 1 week of training in a hotter environment (Buchheit et al. 2011). Heat acclimation to a climatic chamber (35 °C) was also shown to result in significant reductions in final heart rate, Tc and norepinephrine concentrations in eight subjects who performed daily HTT (90 min, walking at 50% maximal oxygen uptake) for eight successive days (Hodge et al. 2013).

### Statistical analysis

Calculations were performed using the software package GraphPad Prism (GraphPad Prism version 5.01 for Windows, GraphPad Software, San Diego, USA). Results were assessed for normality using the D’Agostino and Pearson test.

One-way analysis of variance (ANOVA) and the Friedman test were applied for repeated measures to continuous parametric data and non-parametric data, respectively. The effects of time (Day) and condition (PRE or POST) on continuous variables were determined using two-way ANOVA for repeated measures. Where data were not distributed parametrically but the natural logarithm was parametric, two-way ANOVA was performed using the natural logarithm (log). For data where the log was not parametric, two-way ANOVA was performed only where the Browne–Forsythe test showed no difference in the distribution of data between different times and conditions. Where a significant interaction existed in two-way ANOVA, Dunnett’s multiple comparisons test was applied to assess whether values differed from Day 23 (alpha = 0.05).

A *P* value of < 0.05 was considered significant. Data were summarised as mean ± standard deviation (SD), except where specified as median (range).

## Results

### Completeness of data collection

Two volunteers withdrew due to injuries sustained during non-HTT activities. In a further three volunteers, HRV and/or blood sampling failed at a single time-point (i.e. PRE or POST on 1 day). Thus, PRE and POST study measures were completed successfully at each of the four time points (Day 2, 6, 9 and 23) in 13 volunteers. Partial results from the other five volunteers were excluded from analysis.

### Phase I measurements

Anthropometric and *V*O_2_peak results (*n* = 13) are provided in Table 1.

**Table 1:** United Kingdom baseline anthropometry, *V*O_2_peak and heart rate at *V*O_2_peak for 13 volunteers who subsequently underwent serial HTT in Cyprus

Age (years)	Height (m)	Weight (kg)	Body fat (%)	*V*O_2_peak (mL kg min^−1^)	Peak heart rate (b min^−1^)
23.4 ± 2.8	1.81 ± 0.07	80.55 ± 8.22	16.7 ± 4.7	56.5 ± 7.4	190 ± 9

### Phase II measurements

PRE body mass differed by less than 1% between HTTs. Across all HTTs, Δ body mass from PRE to POST was − 1.60 (− 1.94, − 1.39) kg. There was no difference in Δ body mass between HTTs (*P* = 0.3244), however, Δ body mass per unit Tc rise from HTT decreased significantly with HA, from − 1.1 ± 0.4 kg °C^−1^ on Day 2 to − 1.6 ± 0.6 kg °C^−1^ on Day 23 (one-way ANOVA *F* = 4.662, *P* = 0.0131; post test for linear trend: slope = − 0.17, *P* = 0.0027).

**Fig. 1:**
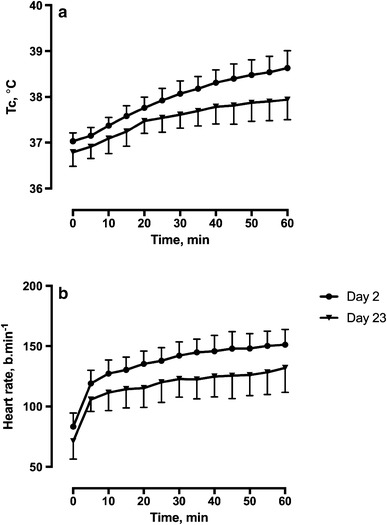
Core temperature (Tc) and heart rate during heat tolerance test on Day 2 and Day 23 in Cyprus (Phase II). Significant difference vs. Day 23, adjusted *P* ^a^< 0.0001, ^b^< 0.0005

Table 1 shows Tc, heart rate and Borg RPE at the beginning and end of all four HTTs. To illustrate the change in physiological strain from the constant-stress model of HTT, Tc and heart rate profiles from during exercise are displayed in Fig. 1.

PRE and POST measures of HRV (log RMSSD, logLF:HF) and biochemical markers of sympathoadrenal activation (plasma nephrines and serum cortisol) are presented in Table 2.

**Table 2:** Core temperature (Tc), heart rate and relative perceived exertion (Borg RPE) at the start (*t* = 0) and end (*t* = 60) of heat tolerance tests in Cyprus

	Day 2	Day 6	Day 9	Day 23
Tc (°C)^a,b,^				
*t* = 0 min	37.0 ± 0.2*	37.0 ± 0.2**	37.0 ± 0.2*	36.8 ± 0.3
*t* = 60 min	38.6 ± 0.4**	38.3 ± 0.4**	38.2 ± 0.4*	37.9 ± 0.4
Heart rate (b min^−1^)^b,c^				
*t* = 0 min	83 ± 11*	75 ± 14	77 ± 12	71 ± 15
*t* = 60 min	150 ± 13**	138 ± 16	135 ± 19	132 ± 20
Borg RPE				
*t* = 0 min	6 (6, 7)	6 (6, 6)	6 (6, 6)	6 (6, 6.5)
*t* = 60 min	13 ± 3**	12 ± 4**	11 ± 2	10 ± 2

Significant (*P* < 0.001) ^a^interaction, ^b^main effect of Day, ^c^main effect of HTT. Significant difference from Day 23, adjusted *P* **< 0.01, *< 0.05

## Discussion

This is the first study to investigate HA-associated changes in HRV alongside biochemical indices of ANS activation. It is also the first to address these changes in relation to structured HTT, and to our knowledge, the first to evaluate sympathoadrenal responses with exercise in humans over an extended period of HA. Novel evidence is presented for autonomic disturbance during the transition between STHA and LTHA, lasting beyond the perturbations observed for classic markers of heat strain. Whereas post-HTT values of heart rate and cortisol were unchanged after Day 2, HRV indices and nephrines indicated ongoing ANS excitability during this period, with values on Day 23 differing significantly for logRMSSD (+ 9.9% vs. Day 6; + 6.4% vs. Day 9), logLF:HF (− 18.6% vs. Day 6) and normetanephrine (− 38.0% vs. Day 9). In addition to providing insights into underlying adaptive processes, these findings highlight the potential added value of HRV measurement in evaluating readiness to work or compete in the heat (Table 3).

**Table 3:** Phase II (Cyprus) measures PRE and POST heat tolerance test assessments

	Day 2	Day 6	Day 9	Day 23
logRMSSD^a,b,c^				
PRE	1.68 ± 0.23	1.65 ± 0.19	1.68 ± 0.20	1.67 ± 0.19
POST	1.34 ± 0.26**	1.42 ± 0.17*	1.46 ± 0.21	1.56 ± 0.20
logLF:HF^a,c^				
PRE	0.38 ± 0.3	0.46 ± 0.16	0.37 ± 0.22	0.51 ± 0.26
POST	0.63 ± 0.3*	0.70 ± 0.24**	0.55 ± 0.32	0.47 ± 0.27
Cortisol (nmol L^−1^)^a,b^				
PRE	413 ± 108	399 ± 149	455 ± 135	382 ± 85
POST	540 ± 202**	327 ± 146	342 ± 162	334 ± 125
Normetanephrine (pmol L^−1^)^a,b,c^				
PRE	228 (176, 272)	257 (175, 280)	291 (253, 329)	267 (216, 297)
POST	687 ± 129**	484 ± 113	574 ± 184**	416 ± 90
Metanephrine (pmol L^−1^)^c^				
PRE	193 (154, 223)	202 (151, 233)	217 (156, 222)	211 (172, 232)
POST	243 ± 90	241 ± 63	243 ± 71	202 ± 50

Significant (*P* < 0.05) ^a^interaction, ^b^main effect of Day, ^c^main effect of HTT. Significant difference from the corresponding value on Day 23, adjusted *P* **< 0.01, *< 0.05. Heart rate variability indices: *RMSSD* root mean square of successive differences, *LF* low frequency, *HF* high frequency

Importantly, these results were observed in an ecologically valid setting of programmed natural HA, with the study’s first aim being to define serial performance status in this setting. International consensus guidelines recommend objective ascertainment of HA progress by assessing physiological responses to exercise in the heat (Bergeron et al. 2012) and one method of establishing this is through noting a lessened heart rate rise during a standard submaximal exercise bout (Racinais et al. 2015). The responses to HTT reported above were commensurate with improved performance capacity from advancing HA status, with Tc and heart rate at end-HTT showing reductions early in STHA and Tc continuing to fall until LTHA. Changes in body mass did not vary by HTT, however, support for a change in thermal thresholds commensurate with HA was evident from the greater loss of body mass per unit rise in Tc from Day 2 to Day 23 (i.e. increased sweating sensitivity to a given increment in body temperature). It can be concluded, therefore, that acclimatisation developed over the course of deployment to Cyprus and that improved capacity to perform in the heat was indeed demonstrated using the selected HTT protocol.

Initial STHA was supported by this constant-stress HTT with the resulting heat strain profile, indicating increasing physiological compensability across the extended course of HA (Fig. 1). At end-HTT, Borg RPE scores indicated an experience of ‘fairly light work’; changes in body mass of 2.0 (1.7, 2.2) % fell within accepted limits; and serum cortisol did not show a significant rise after Day 2, but rather assumed a more diurnal pattern of response with HA. The pattern of plasma nephrine response to HTT also supported the attainment of moderate exercise intensity, sufficient to cause considerable overspill and metabolism of noradrenaline from peripheral nerves (i.e. elevated normetanephrine levels), but not secretion of adrenaline from the adrenal medulla (muted metanephrine response). Thus overall, heat stress from HTT can be considered to have been relatively modest. This highlights the need for a discerning, yet readily measurable, index of HA status when using similar protocols, as relying on a reduction in heart rate alone could potentially provide false reassurance on preparedness for activity in the heat. In contrast, Tc was relatively higher at the end of all HTTs preceding Day 23, highlighting the progressive nature of HA and scope for persistently elevated health risks following arrival to a hot environment.

The concurrent evaluation of HRV and nephrines in the present study provides a unique perspective on the physiological stress buffered by the ANS during this transition from STHA to LTHA. Consistent with the findings of other investigators (Buchheit et al. 2011; Flouris et al. 2014), the measure of parasympathetic modulation (RMSSD) was less affected by HTT as HA progressed. Indeed by Day 23, logRMSSD did not diminish significantly from PRE to POST (adjusted *P* < 0.05). In contrast, responses in the LF:HF ratio peaked on Day 6, indicating relatively greater autonomic modulation at the heart. The LF index is generally associated with baroreflex activation (Heathers 2014) and elevation in the LF:HF ratio has been reported to herald syncope from combined heat and orthostatic stress (Carrillo et al. 2013). Therefore, it is tempting to infer that, in the present study, elevated LF:HF with initial STHA reflected augmented cardiovascular responses in the maintenance of blood pressure.

These changes in HRV occurred alongside biochemical evidence for diminishing sympathetic nervous system activation. Plasma nephrines were assayed in the present investigation, as opposed to circulating epinephrine and norepinephrine, as the latter have a very short half-life of 1–2 min (Peaston and Weinkove 2004) and are so labile they alter in response to a change in posture (Raber et al. 2000). Unlike their parent catecholamines, plasma nephrines are unaffected by venesection and stable, once separated, at 4 °C for 72 h (Deutschbein et al. 2010). Plasma nephrines correlate with their respective catecholamines, but are less susceptible to changes in insulin and glucagon, mental challenge and intraoperative stress from surgery (Roden et al. 2001). It seems reasonable, therefore, to assume that changes in plasma nephrines reflected sympathoadrenal activation during the HTT, rather than being limited to and perhaps confounded by stimuli immediately following HTT. Furthermore, the requirement to protect HRV measures from confounding associated with blood sampling meant that venesection was performed after the 5 min HRV recording period. Thus, assay of the parent catecholamines, which would be expected to have turned over quickly towards their basal values, would have been potentially less informative regarding sympathoadrenal activation over the time course of HTT.

While catecholaminergic responses to exercise in the heat have been reported to reduce over 8 days of HA (Hodge et al. 2013; Maher et al. 1972), the question of whether sustained reductions in norepinephrine might accompany prolonged HA regimens has not previously been addressed. The novel finding of further blunting of normetanephrine responses (LTHA vs. STHA) reported in the present study is relevant to safe performance under heat stress, with the risk of cardiovascular catastrophe from ventricular fibrillation and cardiac arrest understood to increase with enhancement of sympathetic and withdrawal of vagal activity (Brenner et al. 1997). Together, the HRV and nephrine data indicate increasing parasympathetic recovery and progressive blunting of sympathetic nervous system activation with HA, a process that was observed to continue into LTHA. This may relate to reduced competition between the skin microcirculation and the exercising muscles as HA develops (Flouris et al. 2014), with adrenergic withdrawal driving relative bradycardia and vagal dominance over successive exposures to heat stress.

This interpretation of the study results indicates the potential for HRV in non-invasively differentiating responses to exercise and thermal stress, which could perhaps be more revealing than conventional markers of strain during HA. Activation of mRNA transcription from sympathetic signalling during STHA has been proposed to improve cytoprotection during LTHA, through augmented reserves of heat shock protein 70 (Horowitz and Kodesh 2010). Thus, HRV measures could have the potential to be an indirect marker of this process. This would further enhance safety aspects of the assessment of individual ‘readiness’ to work or compete after a period of HA.

This would be relevant to future assessment of HA status, particularly in less developed or more remote settings where methods of monitoring thermal strain may be more limited. Advances in HRV technology have led to increasing miniaturisation, affordability and usability of monitoring/recording devices, while preserving the capability for accurate assessment and data analysis (Boos et al. 2017). ‘Ultra portable’ HRV devices have taken advantage of the robustness and suitability of the RMSSD index to ultra-short recordings (≤ 1 min), as a means of rapidly assessing vagally mediated cardiac control and parasympathetic activity (Esco and Flatt 2014; Flatt and Esco 2016; Nakamura et al. 2015). The relatively low coefficient of variation for RMSSD (~ 12%) is also favourable in comparison with spectral measures of HRV such as LF:HF (Al Haddad et al. 2011), which may improve the signal-to-noise ratio and thus the sensitivity of measurement (Buchheit 2014). While LF:HF may ultimately show too much inter-individual variation to serve as an absolute population ‘cut-off’ marker of ANS modulation with HA, there is scope to track within-subject changes or use alternative HRV measures such as co-efficient of variation (rolling average) (Plews et al. 2012) or sample entropy for this purpose in future work.

In the present study, statistical analyses of both HRV and biochemical data demonstrated main effects of Day and thus indicated an effect of HA, however, the additional interactions between exercise and day of testing likely limit the practical application of these particular variables. The observed interactions are noteworthy, however, as they may themselves point towards or support other evidence for changes in underlying autonomic/endocrine relationships during physiological adaptation. For example, decreased in vitro responsiveness of the atrial beating rate to norepinephrine stimulation has previously reported for laboratory rats acclimated to heat over 5 days, with recovery of responsiveness to heat-naive levels in rats acclimated over 30 days (Horowitz and Meiri 1993). In vivo, catecholamine metabolism can vary with extraneuronal exposure to the enzyme catechol-*O*-methyltransferase (COMT), such that the amount of circulating norepinephrine and normetanephrine metabolite could change according to differential perfusion of COMT-bearing tissues (Bracken et al. 2009; Bracken and Brooks 2010), as may occur with different stages of HA.

These examples and the interactions reported in the above analyses suggest that autonomic biomarkers may perform optimally when ‘reference change values’ are defined to assess serial differences over time (Harris and Yasaka 1983), as may be achieved by comparing one end-HTT sampling point with another, rather than relying upon singleton measurements. Indeed, a serial approach is recommended for other measures intended to assessment of HA, such as the fall in heart rate anticipated to accompany standard exercise bouts (Racinais et al. 2015). Moreover, this reflects understanding of the value HRV may have in evaluating other longitudinal processes, such as training towards competition in elite athletes, whereby intra-individual variation may be tracked over time to detect adaptive and even mal-adaptive responses (Plews et al. 2012). To explore whether absolute or relative referent values of HRV could be developed, either as universal HA markers or for particular populations of interest, a larger investigation than the present study would be required. Unlike the present work, where HRV recording was limited to deployed HTT, this would be expected to include a pre-STHA baseline, i.e. HRV recording in relation to standardised HTT performed some weeks in advance of natural acclimatisation or laboratory acclimation.

The present study has several strengths. Use of HTT conducted at relative exercise intensity ensured that the volunteers, who were of reasonable aerobic fitness, were subject to sufficient exercise and heat stress to demonstrate progressive changes in physiological variables (i.e. acquisition of the HA phenotype). This may not have been possible with a fixed work rate (Epstein et al. 2016). Participants also underwent familiarisation with the protocol in the UK, so the changes in reported variables were less likely to have been influenced by acute psychological reactions to HTT per se. Recording of HRV was performed for the recommended period of 5 min (Task Force 1996), in a standard and reproducible seated position that promoted volunteer comfort and wakefulness (Buchheit 2014). Potential confounding of HRV analysis was reduced further through volunteers abstaining from food and water intake (Lu et al. 1999) for at least 1 h before HTT, during exercise and afterwards (until completion of study measures) and micturating to avoid bladder distension (Fagius and Karhuvaara 1989) before study measures. This also avoided chilled drinks interfering with Tc measurement, which might otherwise have suppressed Tc for 30–60 min post-ingestion (Wilkinson et al. 2008). The moderate intensity of the HTT is expected to have ensured that volunteers remained below the first ventilatory threshold, meaning that vagal activity—rather than respiratory fluctuations—would have contributed to the greater proportion of observed HRV (Buchheit 2014).

Limitations of this study include the reliance on a mixed programme of HA, which was derived from the standard UK military schedule (intended to simulate isothermal HA from progressive increases in distance, load and continuous exercise time), but with the necessary substitution of standard HTTs for programmed exercise on study days. This may have reduced the HA-stimulus on HTT days, as strain fell with successive assessments, and could conceivably have slowed the adaptation rate during STHA. Exercise under this model of constant-stress did, however, serve the purpose of demonstrating adaptation to a fixed requirement for work/performance. It was also important to standardise the exercise intensity of HTT, as this has a graded effect on post-exercise HRV (Parekh and Lee 2005; Michael et al. 2016) and adrenal responses to exercise in the heat also relate closely to relative, not absolute, physiological strain (Wright et al. 2010). Thus, the potential for showing the effects of HA on these variables may have been reduced by adopting a constant-strain HTT. On the other hand, the reproducibility of the selected HTT in achieving a constant-stress model could be challenged on grounds that aerobic fitness may have changed between the assessment of *V*O_2_peak in the UK and LTHA in Cyprus. We are confident that the impact of intra-individual differences in *V*O_2_ was limited, as volunteers followed the same military training protocol pre- and post-departure from the UK (Online Appendix 1), were directly observed to ensure consistency of stepping rate during HTT, and should have improved *V*O_2_peak minimally (~ 5%) through HA-mediated effects on training status (Poirier et al. 2015). It is more likely that following military policy limited improvements in physical fitness per se, with initiation of acclimatisation work delayed until Day 2 in Cyprus (Day 1 being mandated by policy as a rest day, to mitigate any factors such as sleep deprivation that may arise in transit to the hot climate and predispose early heat illness) and volunteers’ usual personal fitness regimens being suspended until Day 10. Nevertheless, this model proved adequate to show physiological progression from STHA to LTHA, and thus infer differences in performance status and risk of thermal injury. In future work, the respective phenotypes could be more fully characterised to elaborate mechanisms of cytoprotection (Assayag et al. 2010) and physiological and metabolic efficiencies (Assayag et al. 2012) that are understood to improve with more advanced HA status.

## Conclusions

Short-term heat acclimatisation was found to be characterised by early reductions in HR and cortisol, whereas LTHA showed diminished excitability by HRV and nephrine measures. These observations indicate changes in autonomic balance that may not be evident from the classic measures recommended for HTT (Racinais et al. 2015), but that may herald the onset of reduced cardiovascular risk, enhanced cytoprotection and improved physiological and metabolic efficiencies specific to LTHA. Further work is required to define the precise phasing of and relationships between ANS/sympathoadrenal and cellular/metabolic changes with advancing HA. This should incorporate assessment by HRV, which could come to offer the prospect of in situ, real-time and non-invasive evaluation of HA status, including attendant performance capacity and health risks.

## Electronic supplementary material

Below is the link to the electronic supplementary material.

Supplemental Material 1. (DOCX 16 KB)

## Abbreviations

ANOVA: Analysis of variance
ANS: Autonomic nervous system
HA: Heat adaptation
HF: High-frequency power
HRV: Heart rate variability
HTT: Heat tolerance test
LF: Low-frequency power
Log: Natural logarithm
LTHA: Long-term heat acclimatisation
MoD: Ministry of Defence
NN: Normal RR
POST: Study measures performed after HTT
PRE: Study measures performed prior to HTT
RMSSD: Root mean square of successive differences between NN intervals
RPE: Relative perceived exertion
STHA: Short-term heat acclimatisation
*V*O_2_peak: Peak oxygen uptake
WBGT: Wet bulb globe temperature

## References

[CR1] Adolph EF (1947). Physiology of man in the desert.

[CR2] Al Haddad H, Laursen PB, Chollet D, Ahmaidi S, Buchheit M (2011). Reliability of resting and postexercise heart rate measures. Int J Sports Med.

[CR3] Armstrong RG, Ahmad S, Seely AJ, Kenny GP (2012). Heart rate variability and baroreceptor sensitivity following exercise-induced hyperthermia in endurance trained men. Eur J Appl Physiol.

[CR4] Assayag M, Gerstenblith G, Stern MD, Horowitz M (2010). Long- but not short-term heat acclimation produces an apoptosis-resistant cardiac phenotype: a lesson from heat stress and ischemic/reperfusion insults. Cell Stress Chaperones.

[CR5] Assayag M, Saada A, Gerstenblith G, Canaana H, Shlomai R, Horowitz M (2012). Mitochondrial performance in heat acclimation—a lesson from ischemia/reperfusion and calcium overload insults in the heart. Am J Physiol Regul Integr Comp Physiol.

[CR6] Bergeron MF, Bahr R, Bartsch P, Bourdon L, Calbet JA, Carlsen KH (2012). International Olympic Committee consensus statement on thermoregulatory and altitude challenges for high-level athletes. Br J Sports Med.

[CR7] Boos CJ, Bakker-Dyos J, Watchorn J, Woods DR, O’Hara JP, Macconnachie L (2017). A comparison of two methods of heart rate variability assessment at high altitude. Clin Physiol Funct Imaging.

[CR8] Borg G (1970). Perceived exertion as an indicator of somatic stress. Scand J Rehabil Med.

[CR9] Bouchama A, Knochel JP (2002). Heat stroke. N Engl J Med.

[CR10] Bracken RM, Brooks S (2010). Plasma catecholamine and nephrine responses following 7 weeks of sprint cycle training. Amino Acids.

[CR11] Bracken RM, Linnane DM, Brooks S (2009). Plasma catecholamine and nephrine responses to brief intermittent maximal intensity exercise. Amino Acids.

[CR12] Brenner IK, Thomas S, Shephard RJ (1997). Spectral analysis of heart rate variability during heat exposure and repeated exercise. Eur J Appl Physiol Occup Physiol.

[CR13] Buchheit M (2014). Monitoring training status with HR measures: do all roads lead to Rome?. Front Physiol.

[CR14] Buchheit M, Chivot A, Parouty J, Mercier D, Al Haddad H, Laursen PB (2010). Monitoring endurance running performance using cardiac parasympathetic function. Eur J Appl Physiol.

[CR15] Buchheit M, Voss SC, Nybo L, Mohr M, Racinais S (2011). Physiological and performance adaptations to an in-season soccer camp in the heat: associations with heart rate and heart rate variability. Scand J Med Sci Sports.

[CR16] Carrillo AE, Cheung SS, Flouris AD (2013). Autonomic nervous system modulation during accidental syncope induced by heat and orthostatic stress. Aviat Sp Environ Med.

[CR17] Chow E, Bernjak A, Williams S, Fawdry RA, Hibbert S, Freeman J (2014). Risk of cardiac arrhythmias during hypoglycemia in patients with type 2 diabetes and cardiovascular risk. Diabetes.

[CR18] Deutschbein T, Unger N, Jaeger A, Broecker-Preuss M, Mann K, Petersenn S (2010). Influence of various confounding variables and storage conditions on metanephrine and normetanephrine levels in plasma. Clin Endocrinol (Oxf).

[CR19] Epstein Y, Yanovich R, Heled Y (2016). Heat tolerance test or race simulation test for return to activity after heat stroke. Med Sci Sports Exerc.

[CR20] Esco MR, Flatt AA (2014). Ultra-short-term heart rate variability indexes at rest and post-exercise in athletes: evaluating the agreement with accepted recommendations. J Sports Sci Med.

[CR21] Fagius J, Karhuvaara S (1989). Sympathetic activity and blood pressure increases with bladder distension in humans. Hypertension.

[CR22] Flatt AA, Esco MR (2016). Heart rate variability stabilization in athletes: towards more convenient data acquisition. Clin Physiol Funct Imaging.

[CR23] Flouris AD, Poirier MP, Bravi A, Wright-Beatty HE, Herry C, Seely AJ (2014). Changes in heart rate variability during the induction and decay of heat acclimation. Eur J Appl Physiol.

[CR25] Harris EK, Yasaka T (1983). On the calculation of a “reference change” for comparing two consecutive measurements. Clin Chem.

[CR26] Heathers JA (2014). Everything hertz: methodological issues in short-term frequency-domain HRV. Front Physiol.

[CR27] Hodge D, Jones D, Martinez R, Buono MJ (2013). Time course of the attenuation of sympathetic nervous activity during active heat acclimation. Auton Neurosci Basic Clin.

[CR28] Horowitz M (2014). Heat acclimation, epigenetics, and cytoprotection memory. Compr Physiol.

[CR29] Horowitz M (2016). Epigenetics and cytoprotection with heat acclimation. J Appl Physiol (1985).

[CR30] Horowitz M, Kodesh E (2010). Molecular signals that shape the integrative responses of the heat-acclimated phenotype. Med Sci Sports Exerc.

[CR31] Horowitz M, Meiri U (1993). Central and peripheral contributions to control of heart rate during heat acclimation. Pflugers Arch.

[CR32] Iellamo F (2001). Neural mechanisms of cardiovascular regulation during exercise. Auton Neurosci Basic Clin.

[CR33] Karlsen A, Nybo L, Norgaard SJ, Jensen MV, Bonne T, Racinais S (2015). Time course of natural heat acclimatization in well-trained cyclists during a 2-week training camp in the heat. Scand J Med Sci Sports.

[CR34] Lu CL, Zou X, Orr WC, Chen JD (1999). Postprandial changes of sympathovagal balance measured by heart rate variability. Dig Dis Sci.

[CR35] Maher JT, Bass DE, Heistad DD, Angelakos ET, Hartley LH (1972). Effect of posture on heat acclimation in man. J Appl Physiol (1985).

[CR36] Michael S, Jay O, Halaki M, Graham K, Davis GM (2016). Submaximal exercise intensity modulates acute post-exercise heart rate variability. Eur J Appl Physiol.

[CR37] Nakamura FY, Flatt AA, Pereira LA, Ramirez-Campillo R, Loturco I, Esco MR (2015). Ultra-short-term heart rate variability is sensitive to training effects in team sports players. J Sports Sci Med.

[CR38] Noe RS, Choudhary E, Cheng-Dobson J, Wolkin AF, Newman SB (2013). Exertional heat-related illnesses at the Grand Canyon National Park, 2004–2009. Wilderness Environ Med.

[CR39] Parekh A, Lee CM (2005). Heart rate variability after isocaloric exercise bouts of different intensities. Med Sci Sports Exerc.

[CR01] Peaston RT, Weinkove C (2004). Measurement of catecholamines and their metabolites. Ann Clin Biochem.

[CR40] Periard JD, Racinais S, Sawka MN (2015). Adaptations and mechanisms of human heat acclimation: Applications for competitive athletes and sports. Scand J Med Sci Sports.

[CR41] Plews DJ, Laursen PB, Kilding AE, Buchheit M (2012). Heart rate variability in elite triathletes, is variation in variability the key to effective training? A case comparison. Eur J Appl Physiol.

[CR42] Poirier MP, Gagnon D, Friesen BJ, Hardcastle SG, Kenny GP (2015). Whole-body heat exchange during heat acclimation and its decay. Med Sci Sports Exerc.

[CR43] Raber W, Raffesberg W, Bischof M, Scheuba C, Niederle B, Gasic S (2000). Diagnostic efficacy of unconjugated plasma metanephrines for the detection of pheochromocytoma. Arch Intern Med.

[CR44] Racinais S, Alonso JM, Coutts AJ, Flouris AD, Girard O, Gonzalez-Alonso J (2015). Consensus recommendations on training and competing in the heat. Sports Med.

[CR45] Roden M, Raffesberg W, Raber W, Bernroider E, Niederle B, Waldhausl W (2001). Quantification of unconjugated metanephrines in human plasma without interference by acetaminophen. Clin Chem.

[CR46] Rowell LB (1990). Hyperthermia: a hyperadrenergic state. Hypertension.

[CR47] Task Force of the European Society of Cardiology and the North American Society of Pacing and Electrophysiology (1996). Heart rate variability: standards of measurement, physiological interpretation and clinical use. Circulation.

[CR48] Taylor NA (2014). Human heat adaptation. Compr Physiol.

[CR49] Tyler CJ, Reeve T, Hodges GJ, Cheung SS (2016). The effects of heat adaptation on physiology, perception and exercise performance in the heat: a meta-analysis. Sports Med.

[CR50] van Ravenswaaij-Arts CM, Kollee LA, Hopman JC, Stoelinga GB, van Geijn HP (1993). Heart rate variability. Ann Intern Med.

[CR51] Wilkinson DM, Carter JM, Richmond VL, Blacker SD, Rayson MP (2008). The effect of cool water ingestion on gastrointestinal pill temperature. Med Sci Sports Exerc 2008.

[CR52] Woods DR, O’Hara JP, Boos CJ, Hodkinson PD, Tsakirides,  Hill NE, Jose D, Hawkins A, Phillipson K, Hazlerigg A, Arjomandkhah N, Gallagher L, Holdsworth D, Cooke M, Green ND, Mellor A (2017). Markers of physiological stress during exercise under conditions of normoxia, normobaric hypoxia, hypobaric hypoxia, and genuine high altitude. Eur J Appl Physiol.

[CR02] Wright HE, Selkirk GA, McLellan TN (2010). HPA and SAS responses to increasing core temperature during uncompensable exertional heat stress in trained and untrained males. Eur J Appl Physiol.

