# Supplementary material for: Heart rate variability and plasma nephrines in the evaluation of heat acclimatisation status
Source: Eur J Appl Physiol. 2017 Nov 10;118(1):165–74. doi: 10.1007/s00421-017-3758-y (PMC5754393; doi:10.1007/s00421-017-3758-y)
Supplement: Supplementary file 1 — Supplemental Material 1. (DOCX 16 KB) [file 421_2017_3758_MOESM1_ESM.docx]

**Online Appendix: Study Programme. Phase 1 (UK) and Phase 2 (Cyprus)**

The study was conducted in two phases. During Phase I, volunteers attended a UK laboratory (Institute of Naval Medicine, southern England) and completed familiarisation HTT. In the following week they undertook directed military training, which was conducted locally over 5 consecutive days (Table A). The WBGT recorded outdoors during working hours was 14.9 ± 2.6 °C. Working dress (full military uniform, boots) was worn with additional torso body armour and a standard external load was carried.

Volunteers then deployed *en bloc* to the Mediterranean island of Cyprus for Phase II of the study. This consisted of structured heat acclimatisation and further directed military training (Table B). Four HTTs were performed in the same climatic chamber during Short Term Heat Acclimatisation (Day 2, Day 6, Day 9) and Long Term Heat Acclimatisation (Day 23). For directed military training in Cyprus (Day 17 to Day 21), the WBGT recorded outdoors during working hours was 28.6 ± 0.8 °C. The training undertaken and military ensemble worn/carried were equivalent to the directed military training experienced in Phase I.

| **TRAINING MISSION** | **Activity** | **Remarks** |
| --- | --- | --- |
| 1 | Patrolling over varying terrain | Patrol load of 40 kg. |
| **Compound Clearance** | | |
| 2 | The section has been tasked to clear a compound of interest. Due to the threat level they enter the compound by scaling the walls using lightweight ladders. | All members of the section will enter the compound using the ladders. Once inside the clearance will require the soldiers to move through confined spaces and crawl through gaps in walls. |
| 3 | The sect finds actionable intelligence in the compound and is re-tasked to clear a suspected enemy compound. | |
| 5 | The section breaches the compound of interest and begins the assault and clearance. There will be 2 enemy within the compound which will be killed. There will be no casualties for the section. | The section will breach the compound by crawling through a hole (simulated explosive entry). They will assault and clear the compound of enemy by conducting short sprints, kneeling and standing between compound rooms. |
| 6 | The section completes the clearance of the compound. | The section will then be re-tasked to clear a vulnerable point (VP) at a track junction. |
| **Room Clearance** | | |
| 7 | Section reaches the VP and begins a visual clearance of the area before conducting the physical clearance. During the VP clearance the section will be attacked by an enemy in a compound approx. 50 - 100m away. | The section will deploy a team in overwatch whilst the remainder of the section conducts the clearance. The overwatch will be conducted by kneeling and laying down. The clearance will require the soldiers to kneel, crawl and lay down. |
| 8 | The section receives orders to assault and clear the compound. The section will breach the compound using lightweight infantry assault ladders to scale the wall. | Once the section has gained entry to the compound they will physically clear each room. The soldiers will be required to operate in a confined space, crawl and utilize kneeling and standing fire positions. |
| **Section Urban Battle Lane** | | |
| 10 | Each section will be given a set route to follow through the urban area in the best possible time. There will be stated tasks to complete along the route. The Battle Lane should take no more than 20min to complete. | Stated tasks will be compound clearance (no enemy), compound clearance (with enemy) and compound wall scaling. Movement will be short sprints of 10 – 50m between compounds and slower deliberate movement within the compounds that require soldiers to crawl, kneel and operate in confined spaces. They will need to cross some ditches and berms that will require small jumps and climbs. |

Table A. Directed military training conducted in UK and Cyprus. Protocol completed in small groups (4 or 5 person ‘sections’) and repeated over 5 consecutive days.

| **Day** | **Dry bulb (°C)** | **Dew point (°C)** | **Programmed Activity** | **Working dress and load** |
| --- | --- | --- | --- | --- |
| Day-1 | 29 | 17 | Rest |  |
| Day 2 | 30 | 16 | HTT-1 | Full uniform, trainers |
| Day-3 | 29 | 18 | 2 x 50 min periods: walk at 6 km/h; rest 15 min; resume walking. | T-shirt and shorts |
| Day-4 | 30 | 19 | 1 x 100 min period: walk at 6 km/hr. | T-shirt and shorts |
| Day-5 | 33 | 15 | 2 x 50 min periods: walk at 6 km/hr; rest 15 min; resume walking. | Full uniform, boots and torso body armour |
| Day 6 | 32 | 20 | HTT-2 | Full uniform, trainers |
| Day-7 | 33 | 15 | 2 x 50 min periods: walk at 6 km/hr; rest 15 min; resume walking. | Full uniform, boots and torso body armour, plus 10kg external load |
| Day-8 | 29 | 19 | 1 x 100 min period: walk at 6 km/hr. | Full uniform, boots and torso body armour, plus 10kg external load |
| Day 9 | 29 | 18 | HTT-3 | Full uniform, trainers |
| Day-10 | 31 | 17 | Rest |  |
| Day-11 to 15 | 34 ± 2 | 14 ± 4 | Light work indoors | Full uniform, boots and torso body armour |
| Day 16 | 33 | 18 | Rest |  |
| Day-17 to 21 | 31 ± 1 | 21 ± 1 | Directed military training | Full uniform, boots and torso body armour, plus external load |
| Day 22 | 30 | 22 | Rest |  |
| Day 23 | 31 | 21 | HTT-4 | Full uniform, trainers |

Table B. Phase II of study programme, conducted in Cyprus following arrival from UK. Midday (1200 h) dry bulb and dew point temperatures are provided from the meteorological station local to the military garrison and adjacent training area.
